# In Situ Transmission Electron Microscopy Investigation of Melting/Evaporation Kinetics in Anisotropic Gold Nanoparticles

**DOI:** 10.3390/ma14237332

**Published:** 2021-11-30

**Authors:** Yunjie Liu, Huanhuan Yuan, Hui Wang, Zhiwei Wang

**Affiliations:** 1 Center on Nanoenergy Research, School of Physical Science and Technology, Guangxi University, Nanning 530004, China; Lyunjie107@163.com (Y.L.); yuanhuanhuan2020@163.com (H.Y.); 2 Beijing Institute of Nanoenergy and Nanosystems, Chinese Academy of Sciences, Beijing 101400, China; 3 Key Laboratory of Aerospace Materials and Performance (Ministry of Education), School of Materials Science and Engineering, Beihang University, Beijing 100191, China; huiwang@buaa.edu.cn; 4 School of Nanoscience and Technology, University of Chinese Academy of Sciences, Beijing 100049, China

**Keywords:** gold nanoparticles, anisotropic, in situ TEM, melting and evaporation

## Abstract

We report on thermal stability and phase transition behaviors of triangular Au nanoprisms through in situ heating transmission electron microscopy. With rising temperature, Au nanoprisms exhibit fluctuating surface reconstructions at the corner regions. When a quasi-melting state is reached at the temperature below bulk melting points, the evaporation is initiated commonly at a corner with low curvature and containing sharp intersection points. The subsequent annealing process leads to the gradual evaporation, which, in the absence of thick carbon coverages, is accompanied by marked shape reconstructions. The thermal stability and evaporation behaviors are not evidently regulated by nanoprism aggregations.

## 1. Introduction

Spatially resolved, real-time observations of heating-induced melting, evaporation or sublimation are of significant importance for fundamentally understanding phase-transition mechanisms and advancing a host of technological applications in, e.g., elemental purification, and controllable synthesis and preparation of nanoscale materials [1,2,3,4,5,6,7]. In situ heating transmission electron microscope (TEM), especially with the introduction of micro-electromechanical system (MEMS)—based heating chips featuring tiny thermal drift and rapid heating rates, has grown as a powerful variable-temperature imaging technique and been widely used in investigating thermal stability and phase transition kinetics of nanostructured materials [8,9,10]. Through direct observations of morphological and structural changes with rising temperature for a variety of metal nanoparticles, the melting/sublimation point depression characteristics were identified and quantified using in situ TEM techniques with reference to theoretical calculations [11,12,13]. Under low-pressure conditions of TEM specimen chamber, Ag nanoparticles exhibit direct solid-gas transitions (sublimations) at the temperature far below bulk melting points [14]. Based on an initial heating observation of Ag nanocubes, {110} was assigned to be the only stable and well-defined surface structure during the sublimation [15], while a further more systematic investigation suggests that the recurring {110} surfaces should be caused by special crystal geometries, and {111} remain to be the lowest potential energy surfaces [16]. A more recent study elucidates that the sublimation of Ag nanoparticles with high surface energy or multiple twin boundaries prefers to proceed nonuniformly, instead of by a uniform pathway as exhibited in those monocrystalline configurations with low surface energy [17]. These in situ TEM observations are generally in accordance with the Kelvin equation, which predicts the increase of vapor pressures with increasing convex surface curvatures [11,12,17,18]. This indicates that higher surface curvatures lead to faster sublimation rates of nanoparticles. For Au, numerous TEM studies have demonstrated that structural transformations occur frequently under thermal heating or even electron beam irradiation, e.g., from icosahedral to decahedral or FCC polyhedral configurations, especially for small nanoparticles [9,19,20]. When Au nanoparticles were heated to well below the bulk melting point, a quasi-melting state is reached with a part of the particles being molten (usually at surface regions), followed by an evaporation starting from the molten regions [9,21].

Herein, we report on in situ heating TEM observations of triangularly shaped Au nanoprisms with aspect ratios of ~1:3, which have considerably larger contact area (with substrates) to volume ratios in comparison with other anisotropic nanoparticles such as nanorods or nanocubes [15,16]. Due to their unique shape, higher surface free energy, and size-dependent physical and chemical properties, etc., Au nanoprisms have potential applications in the fields of catalysis, optics, and biomedicine [22,23,24,25,26]. Our in-situ study, apart from its complementary role to the existing phase transition and kinetics investigations of metal nanoparticles, will also aim to address two emerging questions as follows. One is to determine local structural characteristics of Au nanoprisms when the melting/evaporation is initiated. For anisotropic nanoparticles consisting of flat surfaces, the sublimation was found to usually start from one of low-coordination surface sites (typically corners) [15]. However, it is not yet clear whether the occurrence of evaporation/sublimation is associated with the curvature performances exhibited at these local regions. The other is to reveal the possible influence of nanoparticle aggregation on thermal stability and phase transition behaviors. Previous investigations have been focused on the evaporation/sublimation of individual metal nanoparticles. In fact, the synthetic nanoparticles are often partly aggregated during their growth (especially for high-concentration nanoparticle solutions) or dying processes as driven by the decrease of potential energy [27]. Therefore, we will also carry out heating TEM observations of nanoparticle aggregates to identify whether the evaporation behaviors can be altered by the sintering effect. The phase-transition mechanisms revealed by the in-situ heating TEM imaging should help to the optimized preparation of nanoparticles and nanostructures through, e.g., laser ablation [26].

## 2. Materials and Methods

### 2.1. Synthesis of Au Nanoparticles

Triangularly shaped Au nanoprisms were synthesized based on a one-pot seedless approach as previously reported [28]. Eight milliliters of 0.02 M Cetyltrimethylammonium chloride (CTAC, 97%, from Aladdin, Shanghai, China) was mixed with 1.6 mL deionized water in a small beaker, followed by the addition of 80 μL 25.4 mM HAuCl_4_ (99.995%, from Sigma-Aldrich, Shanghai, China), 75 μL 0.01 M KI (99%, from Tianjin Guangfu technology development, Tianjin, China), 20.3 μL 0.1 M NaOH (from Tianjin Yongda chemical reagent company limited, Tianjin, China) and 100 μL 64 mM ascorbic acid (AA, 99.99%, from Aladdin, Shanghai, China). All the solutions are freshly prepared. When AA was added to the beaker, the color of the reaction solution immediately changed from light yellow to colorless [29]. Then 5 μL 0.1 M NaOH was quickly added under shaking, adjusting the pH value to between 7 and 8. The solution color changed from colorless to purple, and then to blue. The mixture solution was then allowed to stand at room temperature for 10 min in order for the reaction to complete. Take 6 mL of the colloidal solution by centrifugation (10,000 rpm, 10 min) twice, and discard the supernatant. The collected products were dispersed in 3 mL of deionized water and stored in a refrigerator at 4 °C.

### 2.2. Characterization Method

In situ transmission electron microscopy (TEM) characterizations were performed in JEM-2100F (JEOL Ltd., Tokyo, Japan) with an accelerated voltage of 200 kV. To prepare TEM samples, a small drop of the synthesized Au nanoprism solution was drop-cast onto Cu grids covered with amorphous carbon films (for morphological/structural observations) or MEMS—based heating chips. Two specimen-heating holders, Protochips ADURO 100 and DENS solutions Lightning HB, were employed in the in situ TEM study. The constant heating rate of 10 °C/s was set for all the variable temperature experiments. The identical melting/evaporation kinetics of Au nanoparticles are present in the heating experiments conducted with these two holders.

## 3. Results and Discussion

**Figure 1:**
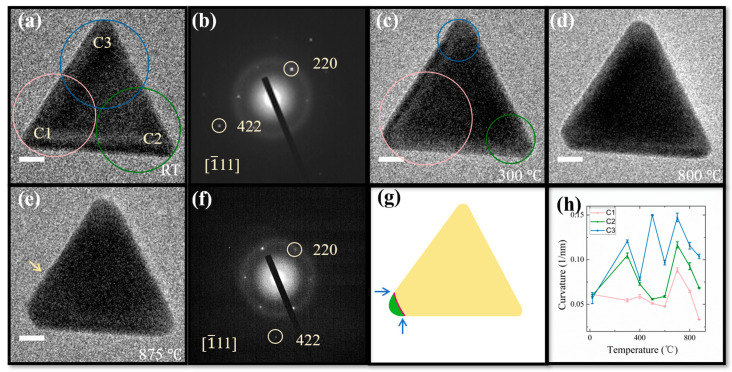
Thermal stability investigation of Au nanoprism. (**a**,**c**–**e**) Bright-field TEM images of a Au nanoprism recorded at room temperature (RT), 300, 800 and 875 °C, respectively. (**b**,**f**) Electron diffraction patterns acquired at the temperatures corresponding to (**a**,**e**), respectively. Crystallographic analyses based on these diffraction patterns manifest that the nanoprism is surrounded by {442} facets. The arrow in (**e**) indicates a slight edge deformation appearing when the quasi-melting point is reached. (**g**) A brief diagram illustrating the intersection formed between flat and spherical surfaces. (**h**) The curvature changes measured at the corners C1, C2 and C3 as marked in (**a**) as a function of temperature. The radius of curvature was determined by drawing a circle that closely fits the short-curved edge as indicated at each of the corners in (**a**,**c**). The error bars are the standard deviations from multiple measurements. Scale bars for (**a**,**c**–**e**): 10 nm.

The synthetic Au nanoprisms generally feature the triangular shape with corner truncation, edge length of 50–70 nm, and thickness of 15–20 nm, with two large {111} surfaces normal to thickness (Supplementary Materials, Figure S1). Figure 1 shows typical in situ heating TEM investigation of Au nanoprisms at various temperatures. Upon increasing the stage temperature from room temperature (Figure 1a) to 300 °C (Figure 1c), there appear a corner rounding at C2 and C3 and a deeper truncation at C1, which leads to the increase and decrease of curvatures, respectively (Figure 1h). The surface reconstruction should be driven by the energy optimization subsequent to the removal of CTAC surfactants around Au nanoparticles as the temperature has now exceeded the melting point of CTAC (~232 °C) [10,27]. A further temperature elevation leads to the fluctuation of curvatures, suggesting a dynamical surface reconstruction behavior presented at all different temperatures. At 875 °C, a quasi-melting state is reached, as evidenced by the slightly curved edge deformation as marked with the yellow arrow (Figure 1e). The electron diffraction pattern acquired at 875 °C (Figure 1f) does not display significant difference from that recorded at room temperature (Figure 1b), except a marked variation of diffraction intensities. This indicates that the crystallographic core structure is retained with only surface regions being in a roughening/melting state, agreeing with previous heating TEM investigations [9]. An example of more marked edge deformation appearing when the quasi-melting state is reached is shown in Supplementary Materials, Figure S2g.

**Figure 2:**
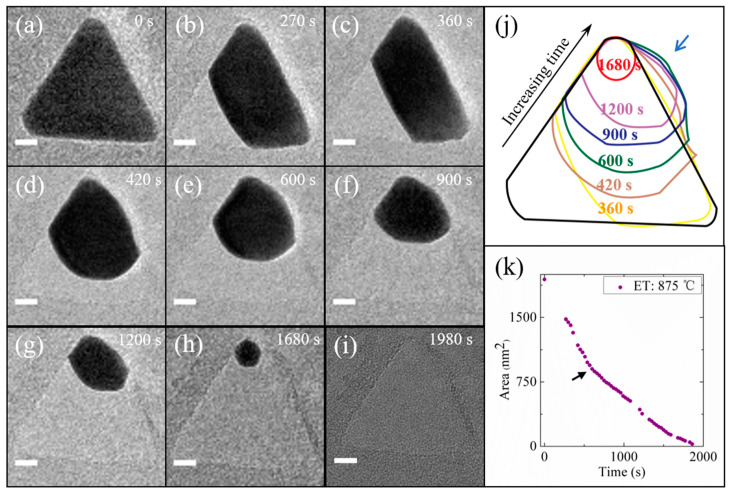
Evaporation kinetics and shape reconstruction. (**a**–**i**) Time-lapse TEM images of the same nanoprism as the one in Figure 1 recorded at 0, 270, 360, 420, 600, 900, 1200, 1680 and 1980 s, respectively, after the temperature was elevated to 875 °C. (**j**) A contour illustrating the shape change of the nanoprism at various heating times (**a**–**i**). The arrow indicates the migration of the nanoprism out of its original position owing to the shape reconstruction. (**k**) The change of projection areas of the nanoprism during the evaporation process. The area change does not exhibit a linear relationship with heating time. The arrow marked at 540 s indicates the transition point of evaporation rates. ET: Experimental temperature. Scale bars for (**a**–**i**): 10 nm.

The evaporation behavior of the nanoprism in Figure 1 was further investigated by recording time-lapse TEM images at the quasi-melting temperature (875 °C). As shown in Figure 2, at 270 s, the corner C1 vanished (Figure 2b), followed by a rapid formation of new, nearly flat {442} surface (Figure 2c). Note that the {442} is not a low-energy surface, and its presence originates from the special crystal geometry effect. This is evidently seen from the subsequent evaporation process, during which the nanoprism gradually developed into a projected circular sector (Figure 2f), and then a nanosphere (Figure 2h) before a complete evaporation (Figure 2i). A brief contour in Figure 2j summarizes the temporal evolution of nanoprism shapes, which manifests that the evaporation-induced shape reconstruction is driven by the minimization of surface energy throughout the entire phase change process. The temporal change of nanoprism projection areas was measured to quantitatively evaluate the evaporation kinetics. As shown in Figure 2k, the area change does not exhibit a linear relationship with heating time. The sharp slope change as indicated with the arrow corresponds to the formation of projected circular sectors. Given that {111} is the lowest-energy surface, the initial evaporation should mainly occur along the {111} surface normal, leaving the nanoparticle thickness basically unchanged. In contrast, there should arise an increase of thickness when the comparatively more spherical nanoparticles are formed during the subsequent shape reconstruction. The evaporation along both in-plane and out-of-plane orientations would be expected for the more spherical nanoparticles consisting of multiple crystallographic faces. In the case of constant evaporation rate in terms of volume change, the decreasing projection-area variations would then arise in the reshaped nanoparticles.

Figure 1h shows that the corner C1 where the evaporation is initiated features the smaller curvature (0.033 1/nm) than the other two (0.068 and 0.104 1/nm for C2 and C3, respectively). In accordance with the Kelvin equation, the lowest vapor pressure is formed at C1, which should thus be more resistant to thermal shock. The fact that we observed the reversible phenomenon could be related to the effect of specific surface geometries of anisotropic nanoparticles. As briefly schematized in Figure 1g, the surface energy should be even higher for the case of lower curvature (red line) at the intersections between flat and spherical surfaces, as marked with the arrows, owing to the emergence of sharper transition. The evaporation could then be more preferably started at these intersection sites when Au nanoprisms are heated to the quasi-melting state.

**Figure 3:**
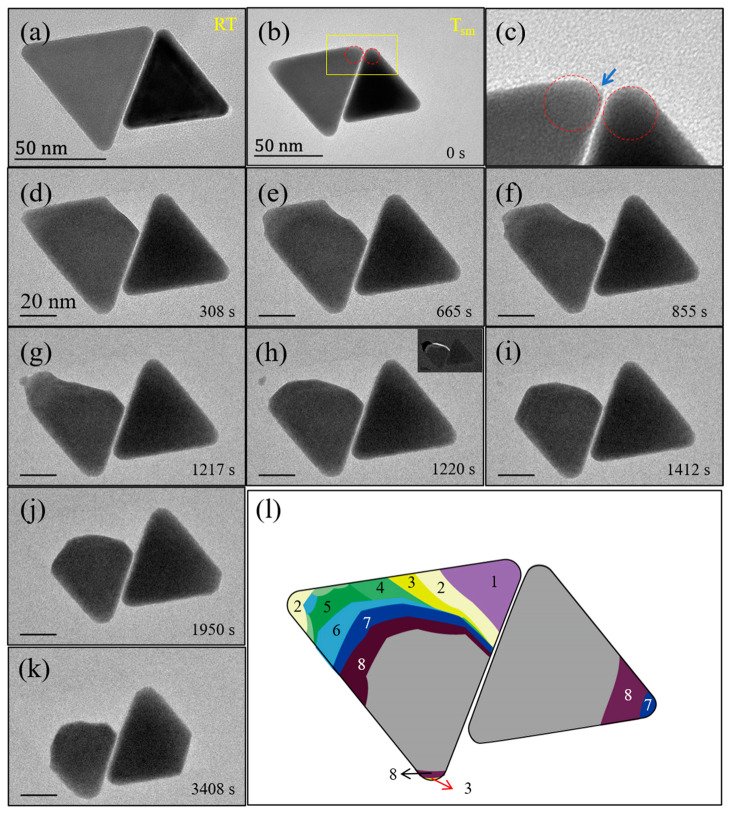
Thermal stability and evaporation pathways of aggregated Au nanoprisms without direct contact. (**a**) TEM image recorded at RT. (**b**,**d**–**k**) Sequential TEM images recorded at 0, 308, 665, 855, 1217, 1220, 1412, 1950, and 3408 s, respectively, after the sample was heated to a quasi-melting state (Tsm). The inset to (**h**) shows a difference image obtained by subtracting (**h**) from (**g**). (**c**) A large view of the image region as marked in (**b**). The arrow indicates sharp intersection between flat and spherical surfaces. (**l**) A contour display for the temporal shape changes of the nanoprism at the heating times (**b**,**d**–**k**). The numbers 1–8 correspond to the shape changes in (**d**–**k**), respectively. Scale bars for (**d**–**k**): 20 nm.

Figure 3 shows typical variable-temperature TEM observations of aggregated Au nanoprisms. It is evident that the two nanoprisms with the distance of ~1.1 nm at room temperature (Figure 3a) remain basically uncorrelated even after the aggregate reaches to a quasi-melting state (Figure 3b), and during the subsequent phase change processes (Figure 3d–k). Their evaporation process exhibits the similar evolution characteristics to that of single nanoparticles. As shown in Figure 3d, the initiation of evaporation happens at a corner near to the interfacial region. Figure 3c shows an enlarged view of the corner region as marked in Figure 3b, in which a sharp transition (as indicated with an arrow) is seen at the corner where the evaporation was started, thus consistent with the observations from single nanoparticles. The evaporation of the nanoparticle is also accompanied with the marked surface reconstruction. This is especially evident in Figure 3g,h. Within only 3 s, the left-side nanoprism changed from an irregular to approximately regular circular sector (projection), as illustrated more clearly by the difference image in Figure 3h inset, and the time-lapse contour of evaporation processes in Figure 3l.

It has been widely reported that nanoparticles are susceptible to coalescence or ripening in liquid solution or solid supports, driven by thermal excitations or electron irradiation [27,30,31,32,33]. Our in situ heating TEM investigations elucidate that the nanoprism aggregates stay unrelated even at the quasi-melting/evaporation temperature. In addition to the size effect (the Au nanoprisms synthesized in this study are generally much larger than the nanoparticles investigated in previous work), the morphology should also play an important role for the experimental observations. Au nanoprisms sit on the Si_3_N_4_ substrates with one of {111} flat surfaces as their interface, so the contact areas are significantly larger in comparison with the case of spherical nanoparticles (point contact, in principle). This consequently makes more difficult the migration of Au nanoprisms on the substrates featuring amorphous structures. In addition, atomic diffusions between two adjacent nanoprisms may be also considerably restricted by the presence of carbon coverages [16]. Although the short-chain organic agent—CTAC (~1–3 nm long) was employed in synthesizing the Au nanoprisms, a layer of carbon substances appears to be still formed after the decomposition of CTAC surfactants under electron irradiation and/or thermal excitation. In terms of our systematic observations, the population distribution of carbon substances is generally not uniform, and, given the much lower atomic number of carbon than that of Au, only those carbon layers with a certain large thickness can be identified by TEM imaging. The presence of thick carbon coverage can also significantly influence the shape reconstruction behaviors exhibited during the annealing process at quasi-melting temperature (Supplementary Materials, Figure S2). Therefore, it could be the multiple factors (size, shape and carbon coverage) that together define the thermodynamics behaviors displayed in aggregated nanoparticles.

## 4. Conclusions

In conclusion, we performed a systematic in situ TEM study of solution-synthesized Au nanoprisms at various temperatures up to quasi-melting points. Upon being heated to 300 °C, the nanoprisms exhibit a dynamic surface reconstruction behavior, typically, corner rounding or deeper truncation, which continuously appears in the process of further temperature elevation. When the sample was heated to quasi-melting temperature, the evaporation usually starts from a low curved corner with sharp intersections. Shape reconstructions are frequently found during the annealing process at quasi-melting temperature, but can be significantly influenced by the presence of thick carbon layers around the nanoparticles. For nanoprism aggregates, no coalescence or ripening behaviors were found between two adjacent nanoparticles even at the quasi-melting temperature, and their thermal stability and evaporation kinetics does not exhibit a marked discrepancy from that of single nanoprisms. These findings provide a valuable reference for the fundamental understanding of evaporation kinetics and contribute to the controllable nanofabrication based on phase change processes.

## Data Availability

The data presented in this study are available on request from the corresponding author.

## Supplementary Materials

The following are available online at https://www.mdpi.com/article/10.3390/ma14237332/s1, Figure S1: Transmission electron microscopy characterization of Au nanoprisms synthesized using the one-pot solution growth technique. (a) A typical low-magnification bright-field TEM image. (b) Electron diffraction pattern recorded for the nanoprism given in the inset. (c) High-resolution image of a nanoprism (processed by Fourier filtering). (d) Statistical edge length analysis of a total of 165 Au nanoprisms. Np: Number of particles. Figure S2: Thermal stability and evaporation pathways of aggregated Au nanoprisms with the presence of carbon coverages. (a–g) TEM images recorded at RT, 300, 400, 500, 600, 700 and 800 °C, respectively. (h–z_b_) Sequential TEM images recorded at the time points indicated in each of the images after the temperature was elevated to 800 °C. Scale bars for all: 10 nm. Note that after the left-side nanoprism has basically vanished, the evaporation of the remaining one proceeds somehow similar to that of single nanoparticles. However, the marked shape reconstruction is not present in this nanoparticle throughout the evaporation process. The elongated shape formed in (v) remains without turning into spheres even at the final stage, in contrast to that presented in the nanoprism in Figure 2. The absence of shape reconstructions should be mainly caused by the presence of amorphous carbon layers formed as a result of surfactant decomposition. For the case of thick carbon coverage, a comparatively darker ring can be visible on the periphery of the nanoparticles, as marked with the arrow in (v). Owing to the presence of carbon rings as supports, the surface energy of elongated nanoparticles (having larger contact area with carbon substances than spherical nanoparticles) could be considerably attenuated, which thus prevents the occurrence of marked shape reconstruction.
