# Supplementary material for: In Situ Transmission Electron Microscopy Investigation of Melting/Evaporation Kinetics in Anisotropic Gold Nanoparticles
Source: Materials (Basel). 2021 Nov 30;14(23):7332. doi: 10.3390/ma14237332 (PMC8658074; doi:10.3390/ma14237332)
Supplement: Supplementary file 1 [file materials-14-07332-s001.zip › materials-1474092-supplementary.pdf]

materials

Supplementary Materials

In Situ Transmission Electron Microscopy Investigation of Melting/Evaporation Kinetics in Anisotropic Gold Nanoparticles

Yunjie Liu [1,2], Huanhuan Yuan [1,2], Hui Wang [3,*] and Zhiwei Wang [1,2,4,*]

[1] Center on Nanoenergy Research, School of Physical Science and Technology, Guangxi University, Nanning 530004, China; Lyunjie107@163.com (Y.L.); yuanhuanhuan2020@163.com (H.Y.)
[2] Beijing Institute of Nanoenergy and Nanosystems, Chinese Academy of Sciences, Beijing 101400, China
[3] Key Laboratory of Aerospace Materials and Performance (Ministry of Education), School of Materials Science and Engineering, Beihang University, Beijing 100191, China;
[4] School of Nanoscience and Technology, University of Chinese Academy of Sciences, Beijing 100049, China
* Correspondence: wangzhiwei@binn.cas.cn (Z.W.); huiwang@buaa.edu.cn (H.W.)

Citation: Liu, Y.; Yuan, H.; Wang, H.; Wang, Z. In Situ Transmission Electron Microscopy Investigation of Melting/Evaporation Kinetics in Anisotropic Gold Nanoparticles. *Materials* **2021**, *14*, 7332. https://doi.org/10.3390/ma14237332

Academic Editor: Oleg Igorevich Lebedev

Received: 5 November 2021
Accepted: 20 November 2021
Published: 30 November 2021

Publisher's Note: MDPI stays neutral with regard to jurisdictional claims in published maps and institutional affiliations.

Copyright: © 2021 by the authors. Licensee MDPI, Basel, Switzerland. This article is an open access article distributed under the terms and conditions of the Creative Commons Attribution (CC BY) license (https://creativecommons.org/licenses/by/4.0/).

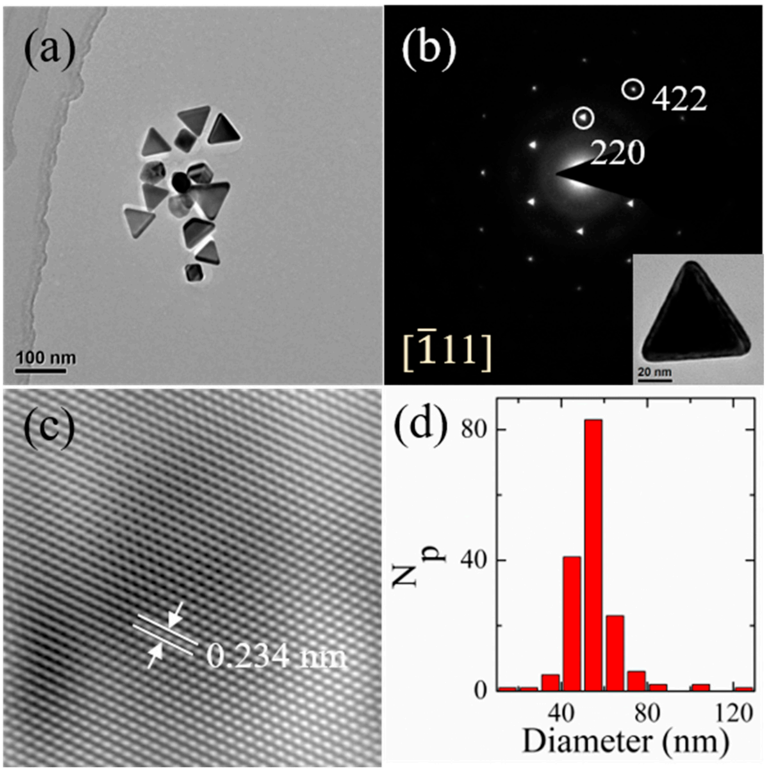

Figure S1. Transmission electron microscopy characterization of Au nanoprisms synthesized using the one-pot solution growth technique. (**a**) A typical low-magnification bright-field TEM image. (**b**) Electron diffraction pattern recorded for the nanoprism given in the inset. (**c**) High-resolution image of a nanoprism (processed by Fourier filtering). (**d**) Statistical edge length analysis of a total of 165 Au nanoprisms. Np: Number of particles.

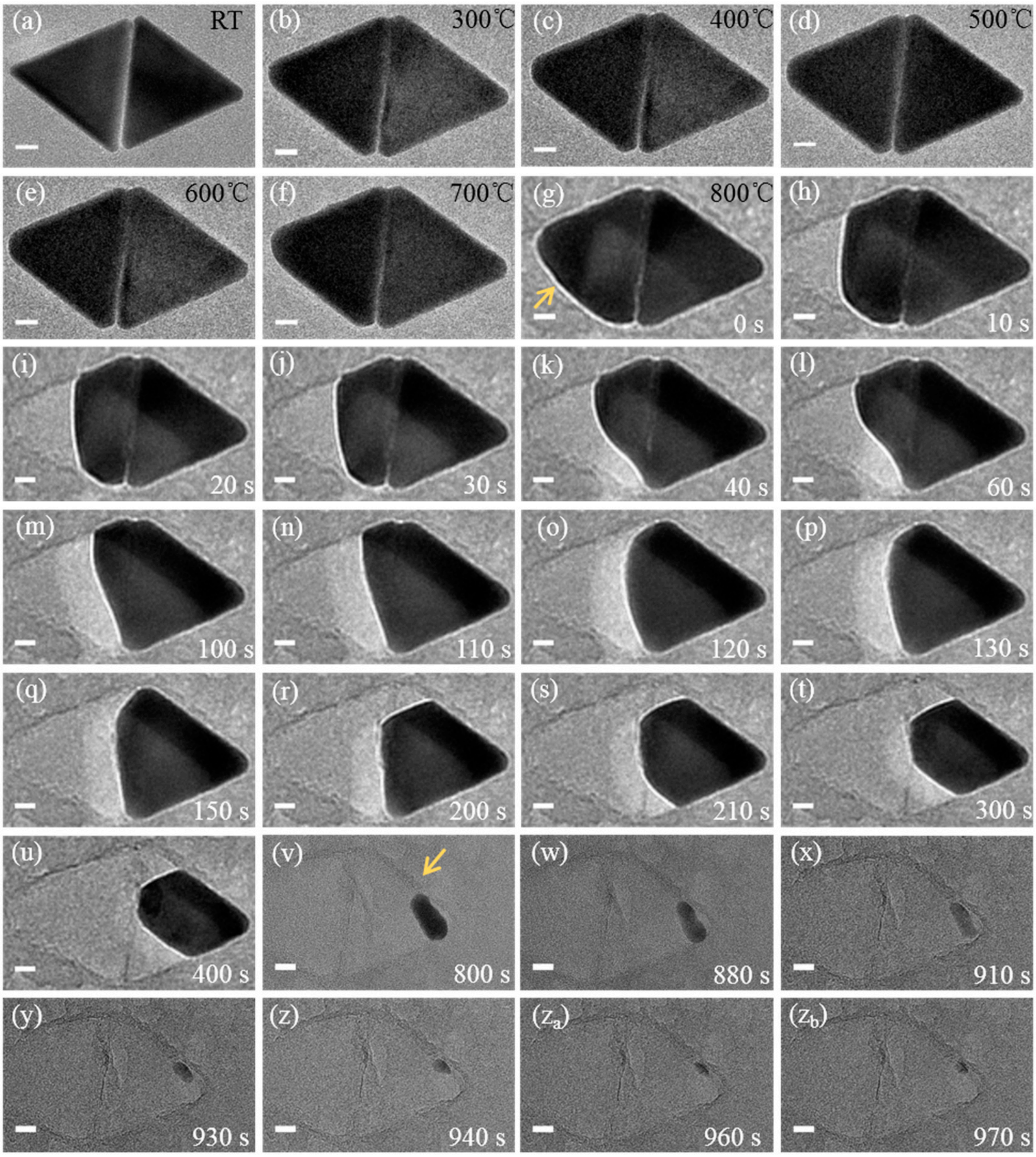

Figure S2. Thermal stability and evaporation pathways of aggregated Au nanoprisms with the presence of carbon coverages. (**a**–**g**) TEM images recorded at RT, 300, 400, 500, 600, 700 and 800 °C, respectively. (**h**–**z$_b$**) Sequential TEM images recorded at the time points indicated in each of the images after the temperature was elevated to 800 °C. Scale bars for all: 10 nm. Note that after the left-side nanoprism has basically vanished, the evaporation of the remaining one proceeds somehow similar to that of single nanoparticles. However, the marked shape reconstruction is not present in this nanoparticle throughout the evaporation process. The elongated shape formed in (v) remains without turning into spheres even at the final stage, in contrast to that presented in the nanoprism in Figure 2. The absence of shape reconstructions should be mainly caused by the presence of amorphous carbon layers formed as a result of surfactant decomposition. For the case of thick carbon coverage, a comparatively darker ring can be visible on the periphery of the nanoparticles, as marked with the arrow in (v). Owing to the presence of carbon rings as supports, the surface energy of elongated nanoparticles (having larger contact area with carbon substances than spherical nanoparticles) could be considerably attenuated, which thus prevents the occurrence of marked shape reconstruction.
